# A late-binding, distributed, NoSQL warehouse for integrating patient data from clinical trials

**DOI:** 10.1093/database/baz032

**Published:** 2019-03-11

**Authors:** Eric Yang, Jeremy D Scheff, Shih C Shen, Michael A Farnum, James Sefton, Victor S Lobanov, Dimitris K Agrafiotis

**Affiliations:** Covance, the Drug Development Division of LabCorp Carnegie Center, Princeton, NJ, USA

## Abstract

Clinical trial data are typically collected through multiple systems developed by different vendors using different technologies and data standards. That data need to be integrated, standardized and transformed for a variety of monitoring and reporting purposes. The need to process large volumes of often inconsistent data in the presence of ever-changing requirements poses a significant technical challenge. As part of a comprehensive clinical data repository, we have developed a data warehouse that integrates patient data from any source, standardizes it and makes it accessible to study teams in a timely manner to support a wide range of analytic tasks for both in-flight and completed studies. Our solution combines Apache HBase, a NoSQL column store, Apache Phoenix, a massively parallel relational query engine and a user-friendly interface to facilitate efficient loading of large volumes of data under incomplete or ambiguous specifications, utilizing an extract–load–transform design pattern that defers data mapping until query time. This approach allows us to maintain a single copy of the data and transform it dynamically into any desirable format without requiring additional storage. Changes to the mapping specifications can be easily introduced and multiple representations of the data can be made available concurrently. Further, by versioning the data and the transformations separately, we can apply historical maps to current data or current maps to historical data, which simplifies the maintenance of data cuts and facilitates interim analyses for adaptive trials. The result is a highly scalable, secure and redundant solution that combines the flexibility of a NoSQL store with the robustness of a relational query engine to support a broad range of applications, including clinical data management, medical review, risk-based monitoring, safety signal detection, *post hoc* analysis of completed studies and many others.

## Introduction

Drug development is becoming significantly more complex and data intensive. Advances in imaging, ‘omics and other technologies have enabled scientists to generate enormous amounts of data and apply them in a clinical trial setting. At the same time, there is a growing need to integrate patient data from non-traditional sources such as electronic health records, biosensors, remote monitoring and fitness devices, social media and other modalities and make it available to study teams using intuitive and actionable visualizations to enhance operational, scientific and medical decision-making. In addition to the difficulty of integrating diverse data sources, there are also operational challenges in determining how to transform data for different use cases, such as medical and safety monitoring, site performance, statistical anomaly detection and submission to regulatory bodies. Particularly, in an environment where these requirements are changing over time and across studies, novel technological solutions are required to manage the complexity of data ingestion and transformation while maintaining performance.

Drawing from our previous work in discovery (1), clinical (2, 3) and outcomes research (4), we have recently introduced an integrated application suite that combines convenient access to data, advanced analytics and seamless integration with established technology to enable comprehensive assessment and mitigation of risk at the study, site and patient level (5, 6). Underpinning these applications is a clinical data repository that supports near real-time acquisition, mapping and integration of clinical trial data from any germane source, comprised of two data warehouses: (i) an operational data warehouse that stores all the operational data and derived metrics and key performance indicators and (ii) a clinical data warehouse (CDW) that stores all the subject-level data. The former is described in the preceding companion article (7). The latter is the focus of the present work.

One of the primary objectives for creating a data warehouse is to provide a consistent and standardized pipeline for data analysis (8). The conceptual model of a data warehouse is to take data from multiple source systems, standardize it, combine it and make it available to a broad range of users through standardized reports, dynamic dashboards and *ad hoc* querying tools to support data exploration and decision-making.

The most challenging aspect of building a data warehouse is the canonicalization of the source data (9). Data from different source systems often have different representations and granularity depending on the design of these systems. In general, the process of designing a data warehouse involves developing a canonical data model, a database schema that best fits that model and the appropriate loading processes to populate it with the source data. Conventional data warehouses work best when the source systems tend to be static with respect to their underlying schemas. By contrast, clinical data are highly variable given the non-standardized nature of clinical trials (10), raising the complexity of the task to the extreme (11). Indeed, the normally difficult work of canonicalizing disparate source data is not a one-time event but needs to be repeated for every trial, and sometimes over time within the same trial (12).

There are two primary approaches that have been used to address this problem. The first is the use of data standards, such as Study Data Tabulation Model (SDTM) or Biomedical Research Integrated Domain Group (BRIDG) (13), for capturing the original data at the source. The second is the adoption of a flexible way of ingesting arbitrary data either via an entity–attribute–value (EAV) model for relational databases or via a NoSQL document store like CouchDB or MongoDB (14). While both methods have had some success, there are important drawbacks that prevent their broader adoption and use.

**Figure 1:**
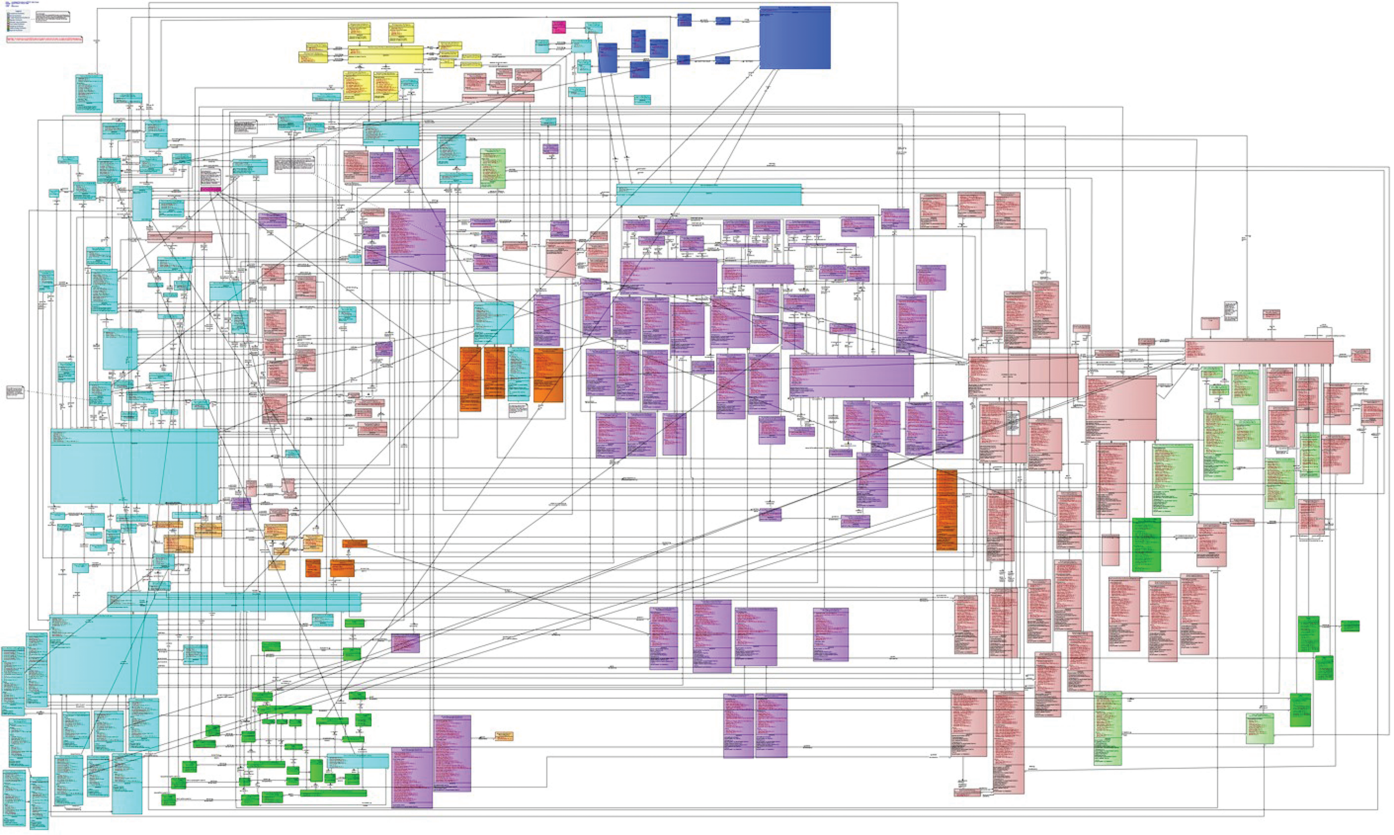
Schematic diagram of the BRIDG model. While this is a comprehensive model for biomedical research, the sheer number of tables and relations between the tables make it difficult to work with in practice. Data managers responsible for the mapping would need to determine for each data source how a row of data would need to be decomposed into this model, putting an inordinate burden on the data standardization process.

The first approach suffers from the fact that data standards lag behind biological and medical science that tends to advance at a faster pace, or they get so unwieldy that become impractical to use and maintain. For instance, the SDTM standard developed by Clinical Data Interchange Standards Consortium (CDISC) (15) organizes the data into a set of ‘domains’ that capture different aspects of a trial. While this model captures the majority of data types encountered in a clinical trial, there is always a small subset of data types, such as exploratory endpoints or measurements, which do not map directly into those predefined domains. To handle these cases, SDTM offers a mechanism for extending the core model via so-called supplemental domains. However, these supplemental domains are essentially an EAV representation of the data, which can dramatically impact performance for many common query patterns, such as when multiple columns need to be returned at once (12). The issue of complexity is exemplified by the BRIDG model that aims to be so comprehensive, that it becomes too convoluted to use in practice (Figure 1).

The second approach of using EAV tables or a NoSQL document framework may offer flexibility in storing arbitrary clinical data but introduces other complications. As mentioned above, EAV models hosted on relational databases suffer significant performance penalties when a wide tabular dataset is requested by the consuming application. On the other hand, NoSQL databases generally do not support relational joins and thus are not as flexible as traditional relational databases in supporting different query patterns. What is desired is a system that offers the flexibility of a NoSQL store for ingesting arbitrary data with the querying efficiency of a relational database.

Here we present a new CDW that combines HBase, a NoSQL column store, with Apache Phoenix, a relational engine built on top of HBase and a user-friendly transformation engine that enables efficient loading of large volumes of incomplete or ambiguous data and defers data mapping until query time. We also discuss how this system facilitates a variety of monitoring, analysis and reporting activities.

## Methods

### Logical architecture

There are two key architectural choices that distinguish our solution. The first is the abandonment of the traditional extract–transform–load (ETL) pattern in favor of an extract–load–transform (ELT) approach (16), and the second is the adoption of Apache HBase and Phoenix as the underlying data store. Depending on the specific use case, it may be sufficient to only utilize one aspect of the design. For example, when the data volumes are not expected to be massive, a traditional relational back end that supports Javascript Simple Object Notation (JSON) or Extensible Markup Language (XML) columns coupled with a dynamic transformation engine could suffice. On the other end, when the incoming data are large but highly standardized, then a distributed NoSQL store with a relational query engine could be used without a transformation engine. The complexity comes from the cases in between, where one has to deal with large incoming volumes of data and multiple (and often evolving) standards, a situation all too familiar to contract research organizations (CROs) like Covance, who must work with multiple clients with divergent needs and operational processes.

The primary driver for adopting an ELT versus an ETL approach stems from the need to coordinate the activities of many different functional groups across multiple organizations and address the gaps in knowledge transfer that arise as a result. This is particularly acute in functional outsourcing, a trial delivery model that is growing in popularity in an effort to curtail costs. For example, while Covance may be contracted to conduct and monitor a clinical trial, the data management activities and the preparation of the submission dataset may be contracted to a third party. In many cases, the transformations from the raw dataset to the target format required to drive downstream analysis and reporting are not communicated to us at the start of the study, and if they are, they may not be sufficient for certain activities like safety monitoring. For instance, the appearance of a particular safety signal may prompt medical monitors to request additional pieces of information, which would require additional data to be queried, transformed and displayed––data for which the transformation rules were not part of the initial contract negotiations between the various parties.

With a standard ETL approach, such changes would require extensive revalidation of the entire process that could lead to production downtime as configurations are migrated or would require two parallel systems to be maintained, one to serve production data and the other to validate the new ETL pipeline before moving into production (17). In an ELT model, the data are ingested in a form as close to raw as possible, and transformations are applied to the data at query time. The obvious advantage of this approach is that multiple sets of transformations (maps) can remain active at any given time, which obviates the need to maintain separate test and production database instances. With an ELT system, it is possible to maintain a test and a production set of transformations within a single database instance. Another advantage is that changes to transformations can be previewed easily and thus the transformation process can be driven by a read–eval–print loop, which allows transformations to be built and tested incrementally. This iterative development process can enable less technical staff to take on many of the tasks currently performed by ETL engineers (18).

As mentioned earlier, the ELT approach does not preclude the use of a relational database. For smaller volumes of data, any relational database management system that supports JSON or XML columns would allow the ingestion of arbitrary data and the transformation of that data at query time. Our decision not to use a relational database is related to scalability. While modern relational databases do have sharding capabilities to help distribute the data across multiple servers, they do not seamlessly handle the addition of new nodes and, when relational joins are desired, most sharded relational databases require additional application-side code to handle joins that span multiple shards.

By contrast, most NoSQL systems scale very well horizontally. When more computational resources are required, one can simply increase the number of nodes rather than buy more powerful hardware. The availability of cloud providers like Amazon Web Services makes scale-up a relatively straightforward and painless task. However, most NoSQL systems traditionally achieved their scalability by not supporting relational joins and instead relying on denormalized schemas (19).

Given the temporal inconsistency in which clinical trial data arrive (i.e. some data streams may be real time while others may arrive at longer time intervals), it is difficult to write an ETL process that can build a fully denormalized representation of the data. Consider, for example, the calculation of ‘study day’, which may require a feed from the interactive voice response system (IVRS) to provide the randomization date for the patient and the visit date that comes from the electronic data capture (EDC) system. Because these systems are typically provided by different vendors, the sponsor or CRO may have different service-level agreements that dictate the frequency of data updates. This process gets even more complicated when these systems use different primary keys, e.g. when the IVRS and EDC use different subject identifiers, and the translation between the two is provided by the sponsor. Some NoSQL-based CDWs have circumvented this problem by focusing only on data from completed trials or ones where all the appropriate denormalizations have occurred prior to loading. The aspirations of the present system were much higher: our goal was to be able to handle all transformations in an online manner and deal with data as they arrive.

Had we utilized a traditional NoSQL system that did not support joins, the data required for this particular study day calculation would need to be pre-joined, and trying to resolve the dependencies would pose a significant challenge. For example, each incoming data point would need to be assessed to determine which derived values would need to be updated. This would greatly hinder the ability to load data in bulk and create a significant bottleneck in the loading process. A system that supports relational logic makes it possible to load the IVRS and EDC data separately in bulk without pre-joining and use the relational engine to combine the two data streams at query time. While this is not impossible to do in a traditional NoSQL database, the problem has already been solved via relational algebra. Given the nature of our incoming data, our goal was to leverage the scalability of a NoSQL system without sacrificing the ability to perform relational joins.

At the time of project initiation, we felt that the only mature solution that satisfied all the requirements was Apache HBase coupled with Apache Phoenix. Briefly, HBase is a column store modeled on Google’s BigTable technology, and Phoenix is a Structured Query Language (SQL) translation engine that converts ANSI SQL into HBase table scans that can be executed in parallel across the cluster. While other SQL-on-Hadoop tools such as Apache Hive or Apache Spark were available, we saw significant performance, ease of use and functionality benefits in favor of Phoenix.

To implement a flexible schema, a user-defined function was developed so that one of the HBase columns would be a JSON column. While HBase and Phoenix natively support dynamic columns, the interplay between secondary indices and these dynamic columns was suboptimal in terms of performance. The key difference is that having a specific JSON column allows the secondary index mechanism to store the row of data directly thus yielding a single lookup, whereas native dynamic columns require a secondary lookup when utilizing secondary indices. This is the only extension that we had to add to the system, and it is compliant with SQL semantics making it possible to select and join columns within this JSON column.

**Figure 2:**
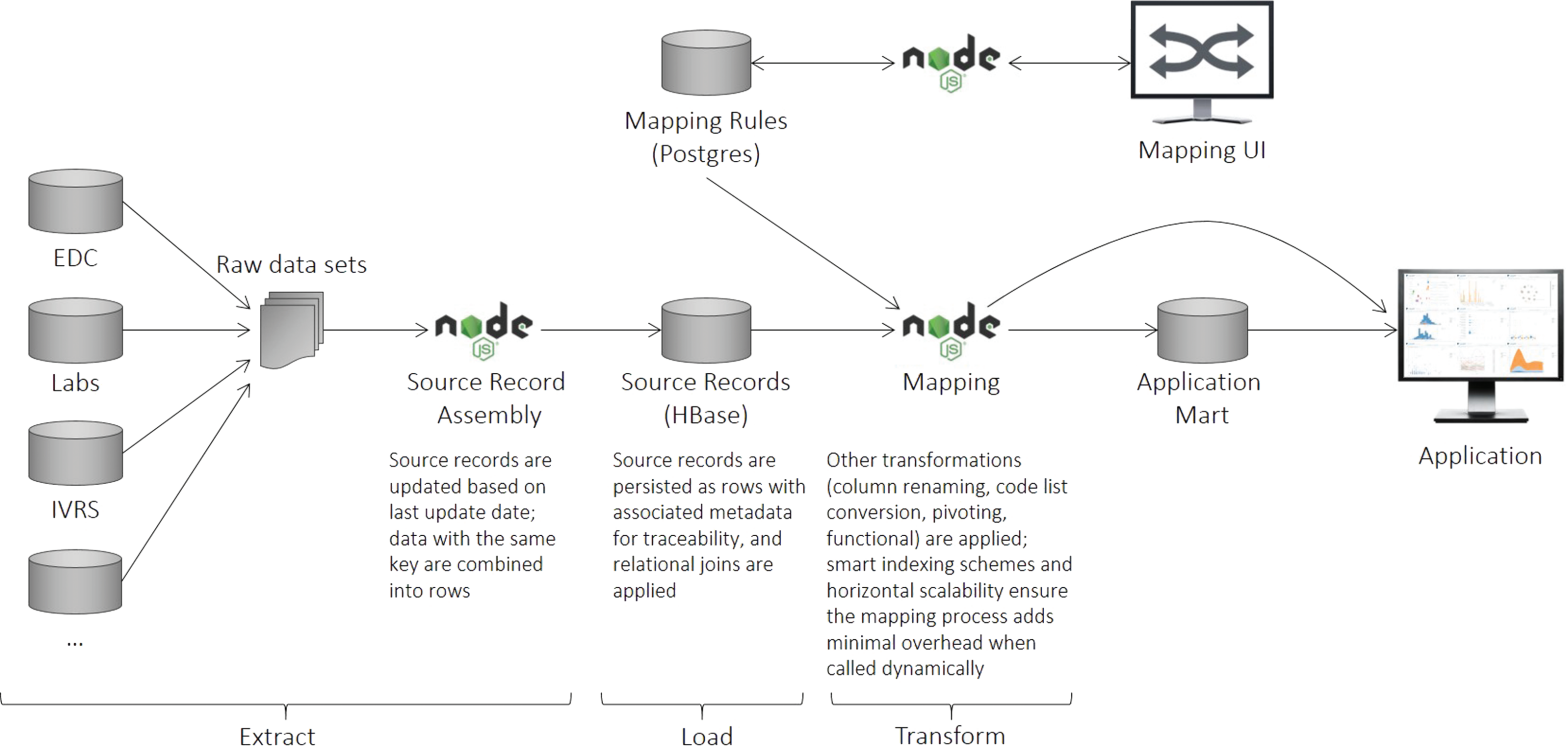
CDW logical architecture. The system consists of an ingest layer that takes raw data files and flattens them into rows of data (records) that can be persisted within HBase. A mapping UI allows transformations to be specified that transform the data upon request. Downstream systems are then able to utilize the data directly or persist them in application-specific data marts to maximize performance.

The ELT process is illustrated in Figure 2 and described in greater detail in the sections below.

### Data preprocessing

CDW ingests the raw data and writes ‘records’ of data to HBase. Here the term ‘record’ refers to a set of related key–value pairs, like a row of data in a Comma Separated Values (CSV) file. Clinical trial data are typically exchanged using a finite number of formats, such as CDISC Operational Data Model (ODM), CDISC Laboratory Data Model (LAB), SAS and CSV. To meet our design objectives, incoming data must first be converted into records. For tabular data this is trivial, but for hierarchical XML representations we must flatten the data before persisting them to the database.

Some file types do not send entire records together; in these cases, we assemble the records during the ingestion process. For example, for ODM data, we consider each ItemGroup to be a record. Unfortunately, the individual elements of an ItemGroup may arrive separately, so during ingestion we assemble records as the ODM data are streamed in. This automation is introduced to make the mapping process easier and more intuitive for the end user. Following the SDTM terminology that divides different types of data into different ‘domains’, the incoming records are grouped into ‘input domains’ that can be thought of as separate tables of data in which the column names are identical for all records (e.g. adverse events and concomitant medications are organized in separate domains).

In addition to being hierarchical, ODM data are transactional, meaning that data from prior files may be referenced in update/delete/restore operations in subsequent files. This means that ingesting ODM data into a data warehouse is not a simple one-way process; it needs to be able to both read and update prior records. We support these kinds of transactional operations in the core of our data ingest engine, making it easy to handle any incremental data formats.

Since the only transformation that we seek to accomplish at the ingestion stage is to either extract rows of data or flatten hierarchical structures into individual rows, the code can be easily extended to handle new data formats as they emerge. This need is infrequent and does not impose a significant operational burden. Once the data have been flattened, we can ingest it into HBase and query it in its raw form using standard ANSI-SQL syntax. Then the raw data must be mapped using a set of transformations.

### Data mapping

Because our input file handlers reduce all of the data into a tabular format, we have been able to reduce the number of mapping transformations into four basic types: column renames, functional transformations, pivoting/de-pivoting transformations and joins.

Column renaming transformations change the name of a column (e.g. from ‘Id’ to ‘SUBJID’). Pivoting/depivoting transformations are structural transformations that convert an EAV table to one where each column represents a different observation, or do the reverse. Join transformations are relational transformations that allow to join data from two different tables. Finally, functional transformations use arbitrary JavaScript functions that operate on individual rows of data. Examples include converting text to upper case, date conversions, code list transformations (e.g. converting gender values from {0,1} to {Male, Female}) and other basic operations like the ones typically implemented through Excel formulas. We also provide the ability to filter data in these transformations.

To illustrate how mapping works, consider two hypothetical data sources, one containing laboratory data from a vendor in the form of a CSV file and the other containing demographic information about the patients in the form of an ODM file.

Lab data (LB) in CSV format containing AST and ALT measurements at visit 1 for subject 1 would come in as follows:

subject,site,visit,testcd,value,dat

0001,1,1,AST,5,2017-10-07

0001,1,1,ALT,6,2017-10-07.

EDC data in ODM format containing age and sex for subject 1, recorded at visit 1 along with the date at which the visit occurred would come in as shown above.

**Table:** 

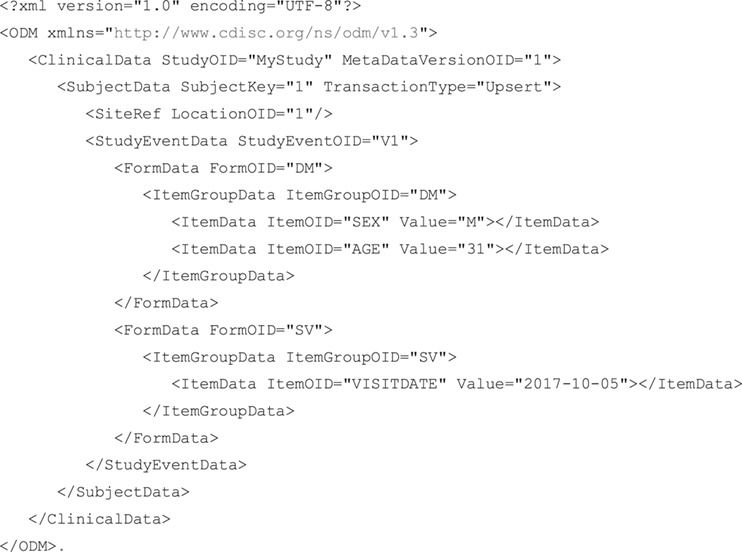

Ingesting these files into CDW would produce four records in HBase:

{

"domain": "LB",

"data": {

"subject": "0001",

"site": "1",

"visit": "1",

"testcd": "AST",

"value": "5",

"dat": "10/07/2017"

}

}

{

"domain": "LB",

"data": {

"subject": "0001",

"site": "1",

"visit": "1",

"testcd": "ALT",

"value": "6",

"dat": "10/07/2017"

}

}

{

"domain": "DM",

"data": {

"StudyOID": "MyStudy",

"MetaDataVersionOID": "1",

"SubjectKey": "1",

"LocationOID": "1",

"StudyEventOID": "V1",

"FormOID": "DM",

"ItemGroupOID": "DM",

"SEX": "M",

"AGE": "31"

}

}

{

"domain": "SV",

"data": {

"StudyOID": "MyStudy",

"MetaDataVersionOID": "1",

"SubjectKey": "1",

"LocationOID": "1",

"StudyEventOID": "V1",

"FormOID": "SV",

"ItemGroupOID": "SV",

"VISITDATE": "2017-10-05"

}

}.

The ‘data’ object in each record comes straight from the raw data files. There would be several problems for someone looking at these data directly:

(i) Different file types use different identifiers, like the subject id being either ‘1’ or ‘0001’ and the visit id being either ‘1’ or ‘V1’. These identifiers need to be reconciled.
(ii) Different file types (and sometimes even the same file types) use different field names for the same concept. In the lab file the visit date is labeled ‘dat’, but in ODM it is labeled ‘VISITDATE’. Field names need to be standardized.
(iii) The contents of these records are determined by the structure of the raw data, but that is not always the structure an end user wants to see. For example, in SDTM, the DM domain should have a RFSTDTC variable containing the date of first drug exposure, but due to the EDC design, that information currently appears in SV.
(iv) Continuing the SDTM example, records in LB should contain not only the date of measurement but also the number of days between DM.RFSTDTC and the date of measurement. This requires doing a join to SV, getting the VISITDATE from the first visit and computing the number of days between SV.VISITDATE and LB.dat.

All of these problems, and more, can be solved during mapping. In this specific example, we would apply several different transformations: (i) rename all the fields according to SDTM; (ii) add the STUDYID to the lab records; (iii) perform joins to make SV.VISITDATE available in other records where it is needed; (iv) use a functional (programmatic) transformation to convert dates to International Standards Organization (ISO) format; and (v) use a functional transformation to calculate LB.DY, the number of days between DM.RFSTDTC and the lab measurement.

The end result looks as follows:

{

"STUDYID": "MyStudy",

"DOMAIN": "LB",

"SUBJID": "1",

"SITEID": "1",

"VISITNUM": "1",

"TESTCD": "AST",

"ORRES": "5",

"DTC": "2017-10-07",

"DY": "3"

}

{

"STUDYID": "MyStudy",

"DOMAIN": "LB",

"SUBJID": "1",

"SITEID": "1",

"VISITNUM": "1",

"TESTCD": "ALT",

"ORRES": "6",

"DTC": "2017-10-07",

"DY": "3"

}

{

"STUDYID": "MyStudy",

"DOMAIN": "DM",

"SUBJID": "1",

"SITEID": "1",

"VISITNUM": "1",

"SEX": "M",

"AGE": "31",

"RFSTDTC": "2017-10-05"

}

{

"STUDYID": "MyStudy",

"DOMAIN": "SV",

"SUBJID": "1",

"SITEID": "1",

"VISITNUM": "1",

"DTC": "2017-10-05"

}.

Mapping clinical trial data to a standardized format necessitates both precision and repetition.

(i) Within a single trial, there is often repetition in form design, such as one different yet structurally similar form for each of a predefined list of important concomitant medications.
(ii) When the same drug is tested in multiple trials, data are often produced in nearly identical formats.
(iii) When the same organization designs multiple trials, they often reuse parts of existing trials (forms, code lists, file formats, etc.), resulting in data that is similar across trials.
(iv) Even across completely unrelated trials, there are similarities. Body mass index is always the ratio between weight and height^2, regardless of the units or the frequency of collection. Race is almost always collected as a series of Boolean values that need to be reduced into either a single race or an indication that the subject has multiple races.

For all of these reasons, transformation of clinical trial data can be done most efficiently by leveraging concepts from programming. Variables, loops, functions and modules allow for more concise and less error-prone mapping.

**Figure 3:**
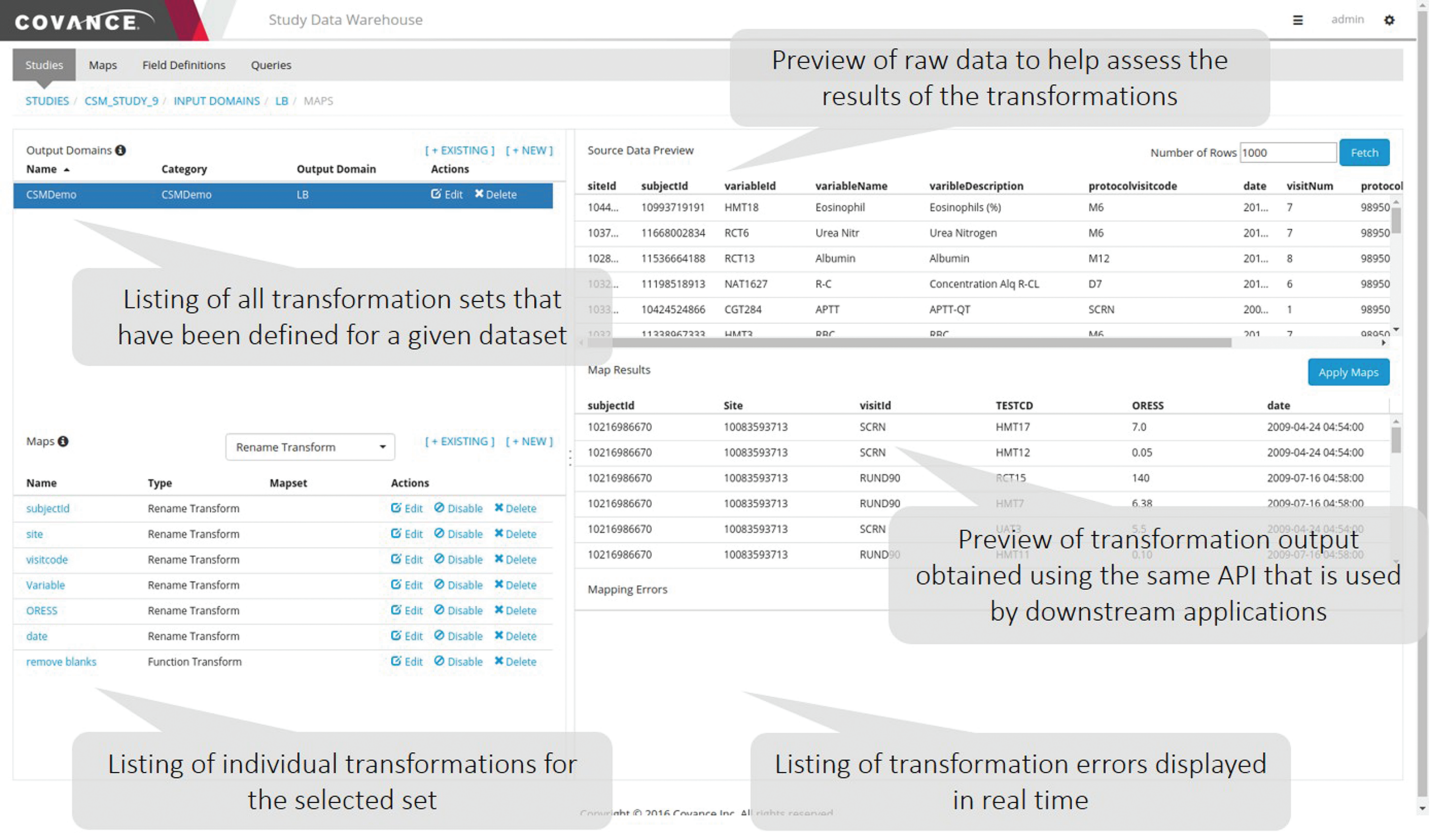
Data mapping user interface. This application allows data managers to view the raw data as it is persisted in the NoSQL store, specify individual transformations to map the raw data to the desired output, execute those transformations sequentially and view the output. Errors identified in the transformation process often highlight ambiguities or missing elements in the specifications.

In most organizations today, mapping clinical data into analysis or submission-ready formats such as SDTM is done by biostatistical programmers using the SAS programming environment. While this is a well-established community within the biopharmaceutical industry, these experts are more scarce and more expensive compared to conventional programmers, and SAS licenses are notoriously costly. As stated above, clinical data mapping involves mundane data transformations (renaming, joins, pivoting, etc.) that do not require any statistical expertise. Parallel to developing a more powerful data warehousing technology, we wanted to create a paradigm shift that would allow us to reduce the cost of clinical data mapping by providing more accessible tools and expanding the pool of qualified candidates for this type of activity. We sought to accomplish this goal both through a graphical user interface (GUI) and an application programming interface (API).

#### Mapping GUI

A representative screenshot of the mapping UI is illustrated in Figure 3. The user starts by selecting a given input domain (as mentioned previously, an input domain is a collection of related data, like a database table) and displaying the raw data associated with that domain. The user then creates an output domain that is a collection of maps (transformations). Each input domain can be associated with multiple output domains either because the data need to be mapped into different forms that are appropriate for different applications or to create multiple output tables for SDTM consumption. One example of the latter is the transformation of data expressed as an EAV table, where different sets of attributes are mapped to different output tables. After an output domain has been defined, a series of individual maps of the five basic types mentioned above are specified one by one and executed in order. The user has the option to preview the data as it has been transformed by the mapping rules at any time. Depending on the results, the user can elect to edit the rules, reorder them or add new ones.

We have found that breaking down the mapping process into these atomic steps and providing a preview window where the results can be examined in real time makes the process easy to follow by clinical data managers who have a good understanding of the data but are not expert programmers. For functional transformations that require some code to be written (e.g. unit conversions, conditional checks, etc.), we have found that basic programming experience is sufficient for writing the majority of the functions required, and individuals who have experience writing formulas in Excel can be retrained to write simple JavaScript functions that operate on a single row of data.

#### Mapping API

While our mapping GUI supports many of these sophisticated operations, we also provide a comprehensive API that, in certain circumstances, can lead to further efficiency gains. Every piece of functionality available to the user in the mapping GUI can alternatively be performed programmatically through our API. The main advantage of this approach is that the tools of modern software development can be used out of the box. Version control manages revision history and collaboration. Unit testing ensures reliability of complex maps. Modules contain reusable functions and variables, allowing very efficient updating of one or more trials simultaneously. Types allow for automatic code completion and prevent invalid maps, which is particularly efficient inside an integrated development environment.

Below we provide an example from the mapping code written in JavaScript for the adverse events domain of a study that we recently mapped, broken down into multiple sections with descriptions.

First, we define the ‘inputDomain’ (the ItemGroupOID from ODM files from an EDC system, in this case) and the ‘outputDomain’ (a short name, like an SDTM domain):

{

inputDomain: 'CORE_DED_AE3001_V1_LOG_LINE',

outputDomain: 'AE',

Then we supply a list of maps. The first maps in the list are simply renaming fields from EDC to more accessible names, done with the ‘field()’ function, which returns a map defining a field:

maps: [

field('CORE_DED_AE3001_V1.I_AETERM', 'TERM'),

field('CORE_DED_AE3001_V1.I_AEGRPID', 'GRPID'),

field('CORE_DED_AE3001_V1.I_AESTDAT', 'STDAT'),

field('CORE_DED_AE3001_V1.I_AESTTIM', 'STTIM'),

field('CORE_DED_AE3001_V1.I_AEENDAT', 'ENDAT'),

field('CORE_DED_AE3001_V1.I_AEENTIM', 'ENTIM'),

field('CORE_DED_AE3001_V1.I_AEONGO', 'ONGO'),

field('CORE_DED_AE3001_V1.I_AETOXGR', 'SEV'),

field('CORE_DED_AE3001_V1.I_AECTCV4', 'CTCV4'),

field('CORE_DED_AE3001_V1.I_AEREL', 'REL'),

field('CORE_DED_AE3001_V1.I_AEREL1', 'REL1'),

field('CORE_DED_AE3001_V1.I_AERELDVC', 'RELDVC'),

field('CORE_DED_AE3001_V1.I_AERELNST', 'RELNST'),

field('CORE_DED_AE3001_V1.I_AERELNSTCM', 'RELNSTCM'),

field('CORE_DED_AE3001_V1.I_AERELNSTDIS', 'RELNSTDIS'),

field('CORE_DED_AE3001_V1.I_AERELNSTOMC', 'RELNSTOMC'),

field('CORE_DED_AE3001_V1.I_AERELNSTPROC', 'RELNSTPROC'),

field('CORE_DED_AE3001_V1.I_AERELNSTNONE', 'RELNSTNONE'),

field('CORE_DED_AE3001_V1.I_AESER', 'SER'),

field('CORE_DED_AE3001_V1.I_AESDTH', 'SDTH'),

field('CORE_DED_AE3001_V1.I_AESLIFE', 'SLIFE'),

field('CORE_DED_AE3001_V1.I_AESHOSP', 'SHOSP'),

field('CORE_DED_AE3001_V1.I_AESDISAB', 'SDISAB'),

field('CORE_DED_AE3001_V1.I_AESCONG', 'SCONG'),

field('CORE_DED_AE3001_V1.I_AESMIE', 'SMIE'),

field('CORE_DED_AE3001_V1.I_AEACN', 'ACN'),

field('CORE_DED_AE3001_V1.I_AEOUT', 'OUT'),

This study includes many entries in the AE form without any data, which we want to filter out since they are not actually adverse events. This is done with the ‘filter()’ function, which returns a map that will filter out any rows where AE.TERM is not defined:

filter('r.TERM != null'),

Arbitrary joins are supported, similar to a relational database. In this case, we need to convert the TERM field to upper case and trim any starting/ending whitespace and then use that processed value as a join key to link to another file containing coded terms (AE.DECOD and AE.BODSYS). ‘copyCol()’, ‘upperCaseTrim()’ and ‘join()’ are all reusable functions that return maps

copyCol('TERM', 'TERM_FOR_JOIN'),

upperCaseTrim('TERM_FOR_JOIN'),

join({

selectedDomain: 'AECoding',

joinCols: ['AETERM'],

colNameTranslations: {

AETERM: 'TERM_FOR_JOIN',

},

selectedColumn: ['I_AETERM_PT', 'I_AETERM_SOC'],

outputColumn: ['DECOD', 'BODSYS'],

}),

A short dictionary is applied with the ‘dict()’ function to convert some values recorded as numbers in EDC to human readable (if a study uses code lists defined in EDC, we can pick them up automatically from the ODM metadata):

dict('SEV', {

1: 'mild',

2: 'moderate',

3: 'severe',

4: 'life-threatening',

5: 'death',

}),

The list of maps can also be a ‘list of lists of maps’, nested arbitrarily deeply. This allows us to define a variable containing multiple maps and reuse it easily. ‘mapsOdmMetadata’ contains some standard fields from ODM files (subject ID, visit ID, site ID, etc.). ‘mapsStdatEndat’ contains a list of maps that convert the AE start and end dates to ISO format, join in the reference start date and compute the study days for the start and end of the adverse event.

mapsOdmMetadata,

mapsStdatEndat,

],

}

This is just a small example of the power of our mapping API, which offers the full capabilities of the CDW mapping engine, including pivoting, unpivoting, arbitrary joins, arbitrary code, reusable functions and more. Furthermore, when another study comes in with a similar AE domain, we are able to quickly abstract the common parts of the above code into a function, ensuring identical and highly efficient mapping.

All of this is also possible through the mapping GUI. The difference is that a skilled user will almost always be more efficient through a programmatic interface, and providing both options offers additional possibilities for increased efficiency and cost reduction.

One challenge in providing both a GUI and API for mapping is interoperability between the two methods. Maps defined with the API can be viewed in the GUI, which is useful for debugging. However, changes made to these maps in the GUI cannot automatically propagate back to the script that created them. When multiple people are working together to map an individual study, communication is important to ensure that either the GUI or the API is the primary tool for mapping that study.

### Data lineage and snapshotting

One particularly powerful feature of HBase is that it maintains data versions for every insert, delete or update transparently and offers a simple mechanism to get the state of the database at any given point in time. Each write to HBase is specified by three pieces of information: a row key, a column name and a timestamp. Any piece of data can be retrieved by this information at any time. Phoenix, through its JDBC connector, can use a CurrentSCN property to specify a timestamp and run queries against prior row values. This allows us to readily access the database state at any point in time without a significant performance penalty and provide data snapshots of the raw data without requiring a separate archival system. This is important because with our ELT framework it is possible to also recreate the mapped data at any time (past or present) without requiring a separate materialization step, a capability that is extremely useful in adaptive trials or other studies involving interim analyses.

Consider the scenario where a data snapshot is generated containing data from patient visits prior to a specific date. An interim analysis for an adaptive trial will often utilize its own special set of transformations, such as the treatment status. However, data errors could still be present when the snapshot was originally taken, and the corrected data may arrive after this cut date. If the snapshots were materialized, updating them would have been an involved process because for all newly corrected data, one would have to update all the snapshots that contained the original erroneous data. Further, because these data arrive as a stream of updates, if one wanted to assess the impact of data changes on the results of the interim analysis over time, multiple time snapshots would need to be generated and persisted. With our ELT approach it is possible to recreate the data state of the interim analysis at any level of temporal granularity.

Regulatory compliance presents another scenario in which it is useful to have a full lineage of both the data and mapping configuration. For example, a regulatory authority may require reproduction of a prior report, even after both the data and mappings have changed. CDW’s ability to recreate the historical state of mapped data makes it trivial to respond to such a request.

To make this possible, it is also necessary to maintain a versioned database of the mapping rules, and we provide such a mechanism in the CDW application database. Every map upon editing creates a new map, and the old version is invalidated. From this audit trail, we can recreate the set of mapping rules that were active at a given time. While storing the full history of mapping rules in a relational database could potentially lead to performance issues if maps are repeatedly edited, we find that after the initial set-up, the maps tend to change relatively infrequently. Given the limited number of maps per domain (<50), the performance hit of fully versioning the maps is not detectable by the end user or any associated ELT processes.

### Data blinding

As described above, the NoSQL framework enables the raw data to be captured as is and presented to downstream applications after going through a series of mapping transformations. One of the maps that we provide is a functional transformation in which a user’s role is used to apply an appropriate blinding transformation. For many applications (see below), data that are mapped from the HBase source record store are written to an application mart, which stores multiple copies of the mapped data, with independent user access rights granted to each of them.

The following is an example of a blinding transformation that can be applied to a single column of data:

if (user.role == 'Blinded' && (Date.now() < new Date('01-01-2019')) {

r['columnToBlind'] = Math.floor(Math.random() * 3)

}

return r;

In this example, if the ‘user.role’ is ‘Blinded’ and the current date is before a date in which blinding should end, a specific column will be fuzzed to contain a random number rather than showing the actual data. This transformation can be extended to also do it on a per-cell basis by altering the ‘if’ statement to take into account other values within that row of data.

The advantage of this approach compared to other methods, such as blinding in the underlying HBase tables through technologies like Apache Ranger, is that more complex rules can be implemented in a manner that is understandable by anyone with basic programming skills, and it also enables automatic unblinding of the data when external conditions change, such as when the database is locked. This allows for seamless transition of the user from blinded to unblinded data for interim database locks.

### Data access and APIs

Following best practices for service-oriented software design, we have developed a set of RESTful web services APIs to facilitate most common data access needs. RESTful APIs ensure secure data transmissions via Hyper Text Transfer Protocol Secure (HTTPS) and bring additional benefits to guarantee performance, scalability, simplicity, modifiability, visibility, portability and reliability of reporting applications. We offer two sets of APIs: one designed to support standard reporting needs and one to support incremental and cumulative data extracts. Both sets of APIs can be utilized for *ad hoc* reporting and could be readily consumed by many commercial reporting tools, such as Power BI, Spotfire, Tableau, Qlik, etc.

### Data marts

As stated earlier, mapping is done dynamically upon data request from an external application or process. The actual mapping process itself is relatively fast, adding only ~10–20% overhead compared to retrieving the raw data directly out of HBase/Phoenix. However, the primary search pattern enabled by this architecture is to retrieve a fully mapped domain, with little support for more general queries. For instance, in the example discussed in the Data mapping section, we would not be able to query for all records where the domain is LB and the DY value (the number of days since randomization) was less than 3 because DY needs to be dynamically computed and with the presence of arbitrary functional transforms, we cannot do the inverse lookups to make relational searches efficient.

**Figure 4:**
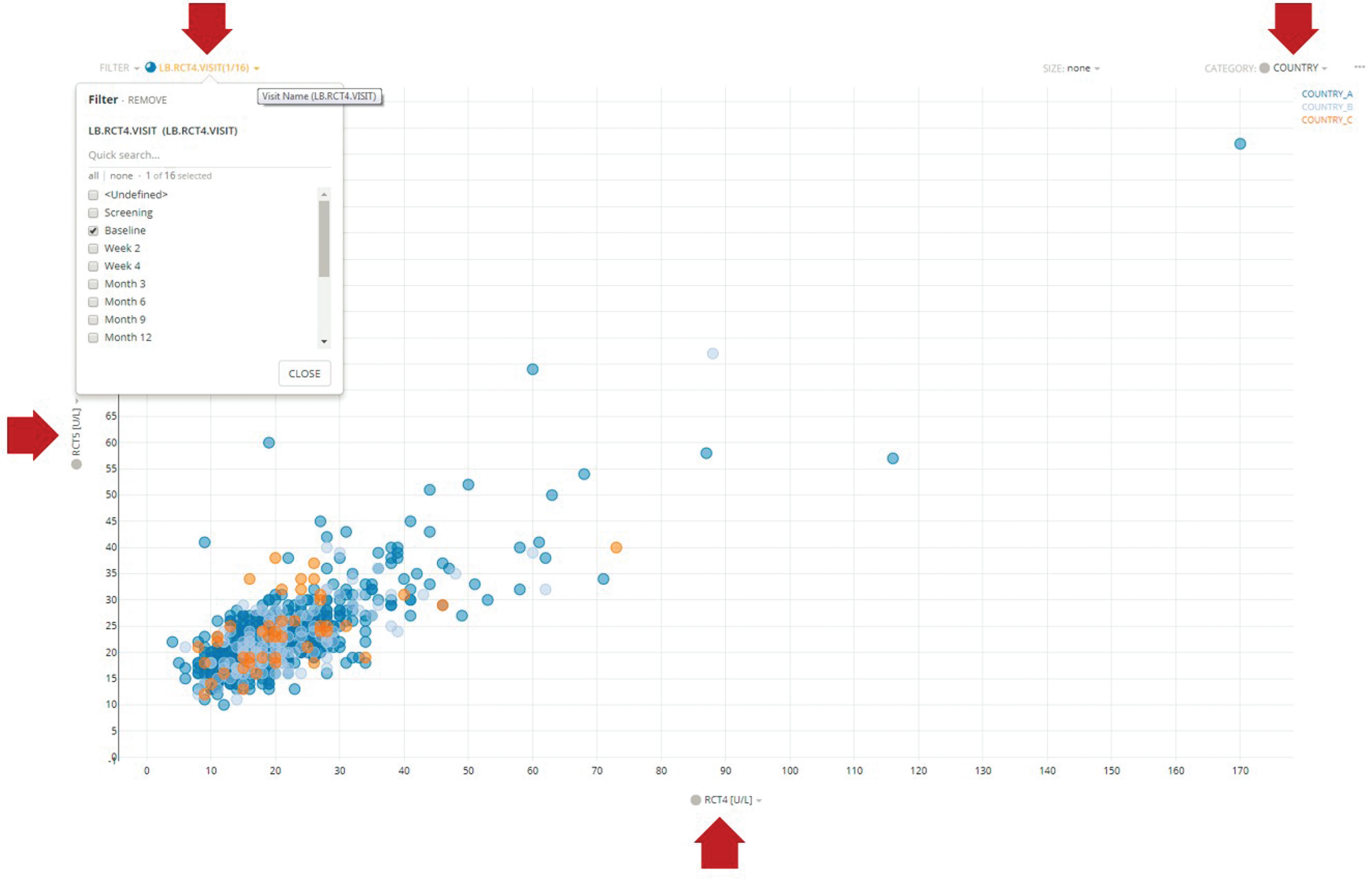
Representative dynamic scatter plot in the Xcellerate Medical Review application. The interface provides four dropdown boxes to allow the use to interactively select which columns of data to use for the X- and Y-axes, as well as for filtering and categorization. In the example shown here, the user has selected RCT4 (ALT) and RCT5 (ALT) for the X- and Y-axes, respectively, country for categorization (so that data points are color coded differently for each country) and the baseline visit as a filter (so that only lab values collected during the baseline visit are displayed). When a different column is selected using these dropdown boxes (e.g. a different lab value for the X-axis), the viewer only needs to retrieve that subset of data from the application-specific data mart and not the entire domain, thus greatly reducing the volume of data that is being transmitted through the network and therefore increasing the responsiveness of the application.

For performance reasons, it is often desirable to create application-specific data marts whose schemas are tuned to the particular query patterns of these applications. While these data marts offer significant performance benefits, mapped data from HBase can still be retrieved at a rate of ~10 000 rows per second. For example, our Xcellerate Medical Review application utilizes a data mart to enable server-side filtering of the data and intelligent transferring of the desired subset of columns on demand, thus greatly improving performance. Consider, for example, the scatter plot illustrated in Figure 4. This dynamic viewer displays two simultaneous columns of data (in this case, ALT and AST) and uses two additional columns for filtering and categorization. The Medical Review application allows the user to dynamically change which columns are displayed on the X- and Y-axes and which are used for filtering and categorization using the dropdown boxes highlighted by the red arrows. When a different column is selected using these dropdown boxes (e.g. a different lab value for the X-axis), the viewer only needs to retrieve that subset of data from the data mart and not the entire domain, thus greatly reducing the volume of data that is being transmitted through the network. This approach speeds up retrieval rates by an order of magnitude (~100 000 rows per second) and makes this application very fast and responsive.

It is worth reiterating that the mapping engine in the underlying HBase NoSQL store remains sufficiently performant for use cases that do not require highly dynamic and responsive UIs, such as statistical analysis and report generation. Fundamentally, there are trade-offs to be made. Data marts do add additional technical complexity and rigidity, and even a frequently updating data mart will exist at some lag relative to the underlying data warehouse; however, data marts facilitate the construction of fast interactive applications that otherwise would not be feasible.

### Physical architecture

**Figure 5:**
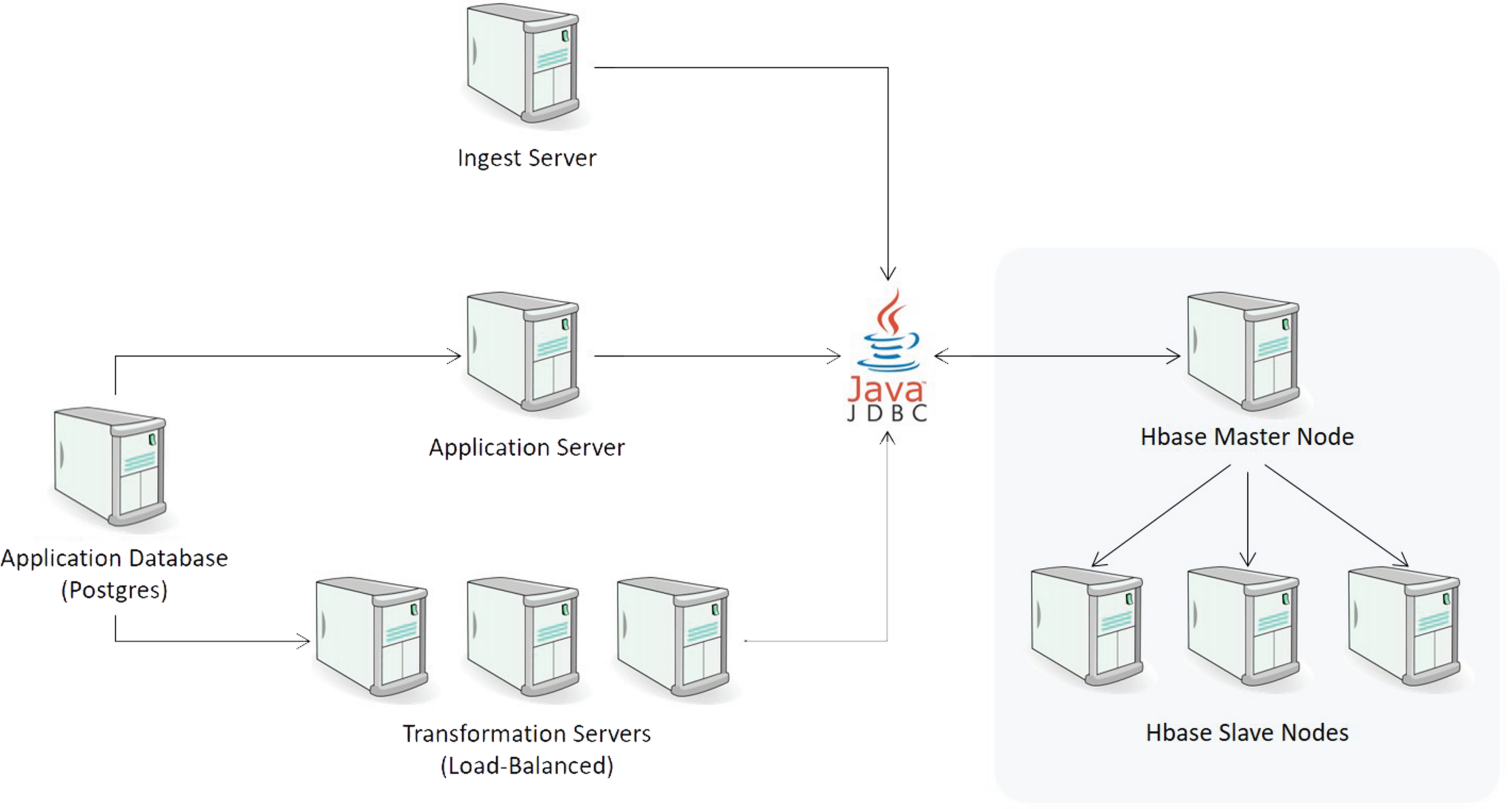
CDW physical architecture.

The physical architecture of CDW is illustrated in Figure 5. Every component of the system was designed to be highly scalable. The ingest layer can be expanded by having multiple ingest servers. If the number of ingest processes overwhelms the current HBase configuration, additional nodes (both master and slave) can be added seamlessly to increase throughput. Similarly, query throughput can be expanded by introducing additional transformation servers. In our current implementation, we dedicate a single transformation server to support the mapping UI and use additional load-balanced transformation servers for any downstream systems that request data from the CDW. This allows our transformation UI to be responsive even under heavy request load from those downstream systems.

The loading layer consists of one or more Node.js ingest servers to parse the incoming files and persist them to HBase. Ingest servers operate in a master/slave configuration where the master node reads a list of loading jobs and distributes them to the slave nodes for execution. This server writes to HBase through Apache Phoenix’s JDBC driver.

One of the main functions of the ingest engine is to maintain proper versioning of the data and ensure that any updates are bumped to a new version. For large amounts of historical data provided in tabular format such as CSV and not event feeds like ODM, the system provides an alternative loader that uses map-reduce to load the data directly in HBase. While this method is significantly faster, it does not maintain proper versioning. Thus, if the same file is ingested N times, there will be N versions of the data within HBase. For one-time import of historical trials, this alternative loader represents a viable option.

Once the data are inside HBase, we can operate on it with standard SQL statements. The underlying schema of our HBase store consists of a series of columns that function as metadata. These include references to the study id, the record id, the id of the ingest job that uploaded the data, the id of the configuration of the ingest job, a flag for soft delete and a timestamp. It also includes a domain column that is akin to a worksheet name in a spreadsheet to handle data streams that contain multiple schemas such as spreadsheets and EDC data feeds. The study id provides a link to an application database that provides additional information.

The record id is a hash that represents the primary key of the record. This key is important because it allows us to determine whether a given record has been updated. HBase automatically maintains versions based upon this primary key and allows the end user to easily query for data that was current at a given date. Therefore, the following query:

SELECT * FROM "sources" WHERE "StudyRef" = 1;

can be made to return data that reflect the database at a given date. This provides a simple mechanism to audit the raw data and show how it changed as data errors were identified and corrected by data managers during the course of the trial.

At this point, the data are at rest in their raw form. However, for these data to be useful in downstream applications, they need to be mapped to a predefined standard or to a client’s specification. As described above, there is a separate web-based mapping application that allows data managers to specify the transformations and preview the results (Figure 3). Though not shown here, one of the advantages of our ELT approach is that it makes it simple for data managers to copy the transformation configuration from another trial and preview the results without having to fully transform all the data. This preview and validation are integral parts of the system and do not require a separate code path. In theory, as the number of trials increases, the likelihood that an appropriate set of transformations already exists increases. These transformations can serve as the foundation for additional automation based on artificial intelligence to assist the data manager by recommending appropriate maps for a new trial (see below). The transformation layer communicates with downstream systems through a RESTful API which, aside from the description of the data requested and the transformations that need to be applied, also includes a parameter to indicate which version of the data (by date) and which version of the transformations should be used.

In the data access layer, upon request, separate Node.js transformation servers query HBase for the desired raw data. With the exception of joins, the transformation servers apply all other data transformations in a streaming manner to take full advantage of the asynchronous nature of Node.js and minimize the amount of memory required to process all the records.

To control the ingest and transformation process, we utilize a central Postgres database that maintains the application state for the transformation UI, along with all the mapping transformations and configurations. Because this database only maintains configuration information (rather than the clinical data itself), the volume of data in it is miniscule. This component is not currently parallelized. The number of writes to this system is minimal, and while the number of reads is higher, it is not of a scale that would require a clustered instance. If that need ever arises, we can utilize the built-in master/slave replication of Postgres to achieve the desired performance.

### Machine learning for semi-automated mapping

In order to simplify the data mapping process, machine learning techniques can be used to suggest possible matches from the canonical data dictionary. In the current implementation of our CDW, the mapping transformation consists of both the field name and the encoding of the underlying data, and thus the use of machine learning could assist in determining which mapping transformation should be applied to a specific column and its content.

Our approach leverages the two primary pieces of information available for a given measurement (the field name and the underlying data itself) to construct a feature vector for each column which, in turn, serves as input to a machine learning algorithm to suggest potential encodings. What complicates this process is the fact that data often come encoded as non-informative codes. For example, we have found that in many cases laboratory measurements are encoded using lab codes that do not provide any hint as to the nature of the test and/or the underlying values [e.g. Covance uses test code 001099 to represent bilirubin (https://www.labcorp.com/test-menu/21111/bilirubin-total)]. Although the data dictionary is eventually provided by the vendor, in many cases it is received at the end of the study, making data canonicalization difficult while the trial is in flight. However, even in those cases, the data values themselves can still be informative.

More specifically, we calculate statistical features of the data both by itself as well as by comparison to a reference dataset. We combine univariate statistics of a given variable such as the mean, standard deviation, skewness and kurtosis along with comparative statistical tests against a curated reference dataset such as *t*-test, *F*-test, interquartile range, Kolmogorov–Smirnov test, etc. These features are combined with text-based information encoded via Bloom filters to construct a feature vector that can be used as input for supervised classification.

We have found that encoding the data into this feature vector represents the most important step and that, given this input, even relatively simplistic machine learning approaches can predict a potential match with a high degree of accuracy. To test this approach, we ran a pilot where we tried to identify our internal Covance lab code from laboratory values received from external labs for three separate trials, each in a different clinical indication (diabetes, HIV and oncology) using a k-nearest neighbors (KNN) classifier along with the feature vector described above. In this experiment, we did not utilize the field names because both Covance and the external labs used non-informative codes. We chose KNN because of its simplicity and the fact that, in addition to the most probable match, it can also suggest other likely alternatives if the primary suggestion is incorrect.

The results revealed that the correct matching code was included in the five nearest neighbors in 75–89% of the cases (89% for the diabetes trial, 88% for the HIV trial and 77% for the oncology trial). Again, this was based on the lab values alone without utilizing any textual information. In many of the cases where the correct match was not in the top 5 hits, there were either multiple highly correlated lab tests having similar ranges and distributions or too many missing data.

We expect that the addition of informative field names in conjunction with more sophisticated machine learning, feature selection and dimensionality reduction algorithms such as neural networks (20), artificial ants (21, 22, 23), particle swarms (24, 25), stochastic proximity embedding (26, 27) and other related algorithms should increase prediction accuracy significantly. This work is currently in progress, and the results will be presented in a subsequent publication.

## Discussion

Given the rate of growth of biological and medical knowledge, data warehouses designed to store clinical trial data must be inherently flexible. Most NoSQL document stores are able to accommodate flexible schemas but are generally designed around fixed query patterns. In clinical trials, the inconsistent cadence of interdependent data feeds and the exploratory nature of many downstream analyses limit the utility of predefined query patterns. As mentioned above, not all of the data will arrive at the same time or in a guaranteed order. Data such as patient identifiers and translation lists may arrive sporadically, making it difficult to maintain all the relationships between different pieces of data in a generalizable and performant manner.

A second imperative is the need for horizontal scalability. As data volumes explode through the use of mobile health, biosensor and other technologies, the ability to expand storage and throughput with minimal effort and disruption becomes an essential requirement. In recent years, traditional relational database management systems, such as Postgres, SQL Server and Oracle, have expanded their capabilities to support data that do not fit within a traditional schema, such as JSON or XML columns. For moderate data volumes, these schema–agnostic columns may indeed suffice. Since our system was intended to manage data for multiple clients and handle data flows from continuous monitoring devices and other emergent sources, we needed a solution that could scale horizontally and redistribute data transparently in order to meet the expected load. Our architecture allows us to start with a small cluster and scale up as data volumes or data access requests increase without having to incur any downtime. Given these two key requirements (flexibility and scalability), we felt that Apache HBase and Phoenix represented the best solution when the project was initiated. By separating the ingestion, transformation and application layers and by utilizing an architecture where each of them could be parallelized in response to load, we were able to meet our objectives.

One of the consequences of adopting an ELT approach is that the raw data and the transformations can be versioned separately. This enables one of the most powerful features of our system, namely, the ability to apply old transformations to new data and new transformations to old data, and recreate the mapped data at any given time point, past or present, without requiring materialized snapshots. Besides the obvious storage benefits, this feature enables use cases that a traditional ETL approach cannot easily meet. For example, in adaptive trials there is often a need to look at data that have been recorded prior to a certain date. However, these data may be erroneous or incomplete for various operational reasons. What is really needed is the ability to see data points that were originally captured prior to a certain date but where cleaned after that date. Additionally, it is often desirable to see how the data change over time in this intermediate state as errors are resolved. With an ELT approach, we can create a set of transformations that filters data by visit date and apply it to the latest version of that data or the version that was current at a given time without having to formally materialize the results.

From an operational perspective, it is also important to be able to transform the data before the final mapping specifications are complete. These specifications tend to evolve over time as errors, oversights or unexpected needs are discovered during the course of the trial. It is much easier to address these problems and communicate between organizations if the output of the transformations can be seen and iterated on in real time. Whether it is due to incomplete or ambiguous specifications, unanticipated signals discovered in downstream applications that require additional transformations or any other reason, being able to accommodate these requests and cascade changes to downstream systems in an agile manner make the process far less frustrating for all parties involved.

The initial version of CDW was based on an ETL approach where the transformed data were persisted in the NoSQL store, rather than being generated on the fly upon data request from a consuming application or process. This design was based on the incorrect assumption that all specifications and data sources would be available prior to the start of the trial. As a result, this version proved of limited value; the need to pre-specify all the transformations was too restrictive and, without live data flowing through the system, it was difficult to resolve ambiguities and issues with data quality. Further, when issues arose during the course of the trial, changing the ETL process was too cumbersome and time-consuming. Finally, we discovered that by analyzing one portion of the data, other questions invariably arose, requiring additional transformations, and we needed a system that was nimble enough to deal with these unforeseen situations. This led to the current design where mappings can be refined in an iterative manner without having to re-materialize old snapshots with updated transformation configurations.

This is perhaps the most important takeaway lesson in our four-year journey that brought us to the current solution. Aside from the specific technology choices, data warehouses intended to support real-time decision-making for ongoing clinical trials must be flexible, scalable and, above all, operationally viable.

## Conclusion

CDW offers a highly scalable, secure and redundant solution that combines the flexibility of a NoSQL column store (Apache HBase) with the robustness and expressive power of a relational query engine (Apache Phoenix). The system enables powerful and flexible mapping of all key data in a repeatable way, allowing for reuse of templates from previous work to minimize manual effort for trial configuration. The NoSQL back end provides scalability and flexibility, allowing seamless parallelization and redundancy for performance and disaster recovery. In addition, new types of records that are expected during the life of the system can be incorporated with only trivial configuration changes. A particularly powerful feature of CDW is the ability to perform data mapping dynamically upon request from an external application or process, which obviates the need to maintain multiple copies or configurations of the data and enables continuous refinement of the mapping specifications as the study is ongoing. By decoupling the versioning of the data and the transformations, we can apply historical maps to current data and current maps to historical data, which greatly simplifies interim data locks and analyses, particularly for adaptive trials. By intelligently automating the data mapping process, the software minimizes the delay in mapping data generated by investigational sites; enables timely review and intervention by monitoring staff; reduces the workload for data management, biostatistics, programming and clinical teams; and brings important practical benefits across a wide range of clinical and translational applications.
